# Phagocytic intracellular digestion in amphioxus (*Branchiostoma*)

**DOI:** 10.1098/rspb.2018.0438

**Published:** 2018-06-06

**Authors:** Chunpeng He, Tingyu Han, Xin Liao, Yuxin Zhou, Xiuqiang Wang, Rui Guan, Tian Tian, Yixin Li, Changwei Bi, Na Lu, Ziyi He, Bing Hu, Qiang Zhou, Yue Hu, Zuhong Lu, J.-Y. Chen

**Affiliations:** 1 State Key Laboratory of Bioelectronics, School of Biological Science and Medical Engineering, Southeast University, Nanjing, People's Republic of China; 2 Nanjing Institute of Paleontology and Geology, Nanjing, People's Republic of China; 3 Guangxi Mangrove Research Center, Beihai, Guangxi, People's Republic of China; 4 Beihai Marine Science and Economy Park, Beihai, Guangxi, People's Republic of China; 5 Department of Neurobiology, Nanjing Medical University, Nanjing, People's Republic of China; 6 Electron Microscopy Research Center, School of Life Sciences, Nanjing Agricultural University, Nanjing, People's Republic of China; 7 Department of Pathology, Nanjing Drum Tower Hospital, Affiliated Hospital of Nanjing University Medical School, Nanjing, People's Republic of China

**Keywords:** phagocytic intracellular digestion, *Branchiostoma*, digestive tract, diverticulum, gut, gene expression

## Abstract

The digestive methods employed by amphioxus (*Branchiostoma*)—both intracellular phagocytic digestion and extracellular digestion—have been discussed since 1937. Recent studies also show that epithelial cells lining the *Branchiostoma* digestive tract can express many immune genes. Here, in *Branchiostoma belcheri*, using a special tissue fixation method, we show that some epithelial cells, especially those lining the large diverticulum protruding from the gut tube, phagocytize food particles directly, and *Branchiostoma* can rely on this kind of phagocytic intracellular digestion to obtain energy throughout all stages of its life. Gene expression profiles suggest that diverticulum epithelial cells have functional features of both digestive cells and phagocytes. In starved *Branchiostoma*, these cells accumulate endogenous digestive and hydrolytic enzymes, whereas, when sated, they express many kinds of immune genes in response to stimulation by phagocytized food particles. We also found that the distal hindgut epithelium can phagocytize food particles, but not as many. These results illustrate phagocytic intercellular digestion in *Branchiostoma*, explain why *Branchiostoma* digestive tract epithelial cells express typical immune genes and suggest that the main physiological function of the *Branchiostoma* diverticulum is different from that of the vertebrate liver.

## 1. Introduction

Every organism requires energy to survive, and most heterotrophic unicellular organisms phagocytize food particles directly and break them down via intracellular digestion [1]. Phagocytic intracellular digestion is the evolutionary cornerstone of both the digestive and immune mechanisms of multicellular animals [2–6]. Currently, however, phagocytosis in deuterostomes is seen primarily as an immune process, and research into it focuses on the biological mechanisms used to eliminate pathogens, clear up cell debris and present antigens [7]. It is commonly accepted that animals, especially deuterostomes, digest food using only extracellular digestion, which is the process of breaking down large food particles into small, water-soluble absorbable molecules by digestive enzymes in the lumen of a digestive organ [8]. In 1937, however, van Weel observed that, in addition to general extracellular digestion, the digestive tract of *Branchiostoma*, especially its protruding part, the diverticulum, can phagocytize food particles directly. He, therefore, suggested that both extracellular and intracellular digestion can occur in *Branchiostoma* [9]. Nevertheless, because autolysis by intracellular digestive enzymes tends to occur during sample fixation, phagocytic intracellular digestion in the *Branchiostoma* diverticulum is difficult to observe when using the typical fixatives used for light and election microscopy [10–14]. In addition, because the *Branchiostoma* diverticulum has been regarded as the homologue of the vertebrate liver for more than 150 years—since it was first hypothesized by Müller in 1844 [15]—it is difficult to accept that the living animal, like the chordate ancestor, mostly could use its ‘liver’ to ‘eat’ food directly [10,12].

Recent studies of the *Branchiostoma* digestive tract focus on tracing the origin of the vertebrate immune mechanism [16–27], but neglect its original digestive function. For instance, though variable region-containing chitin-binding proteins (VCBPs) have been shown to play important roles in the adaptive-like immune response [16–19] in *Branchiostoma*, there has been little investigation into why the VCBP genes are expressed in endoderm-derived epithelial cells of the digestive tract but not in mesoderm-derived professional immune cells. Consequently, determining the biological characteristics of the epithelial cells in the digestive tract, especially those in the diverticulum, is key to understanding the role of phagocytic intracellular digestion in *Branchiostoma*, and could shed light on the origins of the vertebrate immune mechanism and liver.

**Figure 1.**
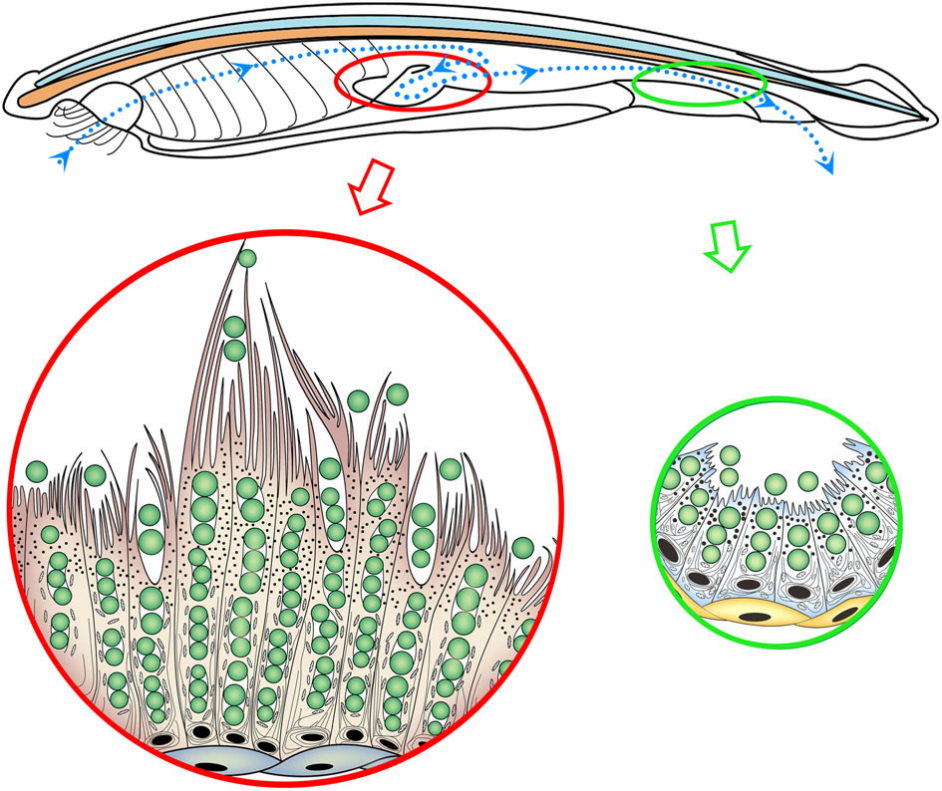
Phagocytic epithelia of the *Branchiostoma* digestive tract. The epithelial cells of the diverticulum and hindgut can phagocytize food particles directly, and the phagocytic capability of diverticulum cells is greater than that of the hindgut epithelial cells.

Here, by using an improved method of tissue fixation for transmission electron microscopy (TEM), we show that *B. belcheri* epithelial cells, especially those in the diverticulum, can phagocytize food particles directly (figure 1). The gene expression profile of these cells reveals that they can support both digestive and immune-like functions by expressing many kinds of immune genes and varying types and amounts of digestive enzymes. Interestingly, qRT-PCR results demonstrate that the gene expression profile of the cells can present two different states: after 3 days of starvation, they accumulate many kinds of digestive enzymes, as in vertebrate digestive cells, whereas, when the animal is sated, they also express immune genes in response to stimulation by exogenous food particles. Our results shed light on the context in which phagocytic intracellular digestion occurs in *Branchiostoma*, and suggest why the epithelial cells of the *Branchiostoma* digestive tract can express immune genes.

## 2. Material and methods

### (a) Animal and embryo procurement

Adult *B. belcheri* were collected from Zhanjiang Bay, Guangdong, China, during the summer breeding season (20 June–10 July, 2008–2015), and fed with cultured mixed algal powder including *Spirulina platensis*, *Isochrysis galbana*, *Chlorella vulgaris* and diatoms, etc., while being kept at 28–32°C at the Beihai Marine Station of Nanjing University, Guangxi, China. Wild samples are assumed to be sated because of the plentiful algal resources in Zhanjiang Bay [28]. Gametes were acquired by heat stimulation at 35–37°C, and then fertilized in filtered seawater. The embryos and larvae were cultured at 30 ± 2°C.

### (b) Transmission electron microscopy and scanning electron microscopy

We sought a way to improve sample fixation so as to be able to visualize phagocytic intracellular digestion in the *Brachiostoma* digestive system. We found that initial fixation including glutaraldehyde is insufficient to fix the phagocytic epithelial cells of the *B. belcheri* digestive tract. Here, we used MEMPLA (0.1 mol l^−1^ morpholinopropanesulfonic acid, 2 mmol l^−1^ EGTA, 1 mmol l^−1^ MgSO_4_, 4% paraformaldehyde, 0.5 mol l^−1^ NaCl, pH 7.5, freshly mixed) to fix the *B. belcheri* digestive tract at 4°C for 12 h, prior to transfer to 2.5% glutaraldehyde in 0.02 mol l^−1^ PBS at 4°C. Other steps for TEM viewing and scanning electron microscopy (SEM) experiment were as previously described [29–31].

### (c) The construction, sequencing and analysis of the full-length cDNA transcriptome library

Diverticula were dissected from 1-year-old adults and pooled from 100 individuals to ensure that enough RNA could be obtained (greater than 1 mg) for a full-length cDNA transcriptome library (hereafter ‘Full’). The total RNA was isolated with TRIzol LS Reagent (Thermo Fisher Scientific, 10296028) and treated with DNase I (Thermo Fisher Scientific, 18068015). The high-quality mRNA was isolated with a FastTrack MAG Maxi mRNA Isolation Kit (Thermo Fisher Scientific, K1580-02). To ensure that the results of Full reflect the natural state of diverticulum epithelial cells, we used the ‘technical route without normalization’ when constructing the library. The construction of Full was performed with a SuperScript Full-Length Library Construction Kit II (Thermo Fisher Scientific, A13268) with pDONR222 Vector according to the manufacturer's instructions (electronic supplementary material, figure S6).

The recombination pDONR222 vectors were sequenced with the Sanger method in a Thermo Fisher Scientific 3730xl DNA Analyzer using M13 forward primer; 15 000 vectors were sequenced, and 14 976 efficient expressed sequence tags (ESTs) were obtained. The redundancy statistic of the sequencing results is approximately 70% and reached the plateau phase. All the genes with Clustered-EST counts ≥10% were sequenced throughout from 5′ end to poly(A)^+^.

The unigenes were analysed using ORF finding, expression profile analysis, NCBI, SWISSPROT, KEGG, COG, Interpro and GO (electronic supplementary material, figure S7 and tables S3–S6). The annotated tissue-specific genes were verified by conserved homologous sequence using ClustalW and neighbour-joining trees using Mega6 with 1000 bootstraps.

### (d) qRT-PCR assays

The qRT-PCR assays for determining the gene expression profile of the diverticulum epithelial cells include all the tissue-specific unigenes in Full (electronic supplementary material, table S2). From 1-year-old adults, we dissected the empty epithelial cells of the diverticulum (ED), natural sated epithelial cells of the diverticulum (SD), natural sated gut (SG), empty gut (EG), as well as pharyngeal bar (PB) and notochord (NT) cells as contrast samples. The empty samples were from animals that were starved for 3 days to ensure there were no residual algal cells in the epithelial cells of the digestive tract and that the gut had time to excrete all faeces completely.

The total RNA isolation was processed in the same way as the construction of Full. The two-step reverse transcript reaction Mixes I and II were prepared for the qRT-PCR reaction as follows. Mix I is a 12 µl system for 2 µg total RNA (adjusted to 5 µl), 0.5 µl of 50 µmol l^−1^ Oligo (dT) Primer (Thermo Fisher Scientific, AM5730G); 0.5 µl of Random Primer (Thermo Fisher Scientific, 48190011); 1 µl of 10 mmol l^−1^ dNTP Mix (Thermo Fisher Scientific, 18427013) and 5 µl of Nuclease-Free Water (Thermo Fisher Scientific, AM9938). Mix II is a 20 µl system consisting of 1 µl of SuperScript III RT at 200 U µl^−1^ (Thermo Fisher Scientific, 18080093), 4 µl of 5X First-Strand Buffer (Thermo Fisher Scientific, 18080093), 2 µl of 0.1 mol l^−1^ DTT (Thermo Fisher Scientific, 18080093); 1 µl of ribonuclease inhibitor at 40 U µl^−1^ (Thermo Fisher Scientific, 10777019) and 12 µl of Mix I. We kept Mix I at 65°C for 5 min, then put it on ice for 1 min and kept Mix II at 25°C for 5 min, 50°C for 1 h, 70°C for 15 min and on ice for 1 min. This cDNA can be stored at −20°C for six months. The PCR reaction is a 20 µl system performed according to the experimental standard of the manufacturer using the SYBR Green (Thermo Fisher Scientific, S7567) method.

The primers were designed online using Primer3 and NCBI Primer-BLAST, and the melt temperature (*T*_m_) was set at 60 ± 0.5°C. All the primers were screened by electrophoresis and PCR product sequencing. The PCR products were purified with an E.Z.N.A. Ultra-Sep Gel Extraction Kit (Omega Bio-tek, D2510-01) and inserted into pMD18-T Vector (TaKaRa, 6011), then sequenced with the Sanger method using M13 forward primer. Matlab (MathWorks, USA) was used to process the raw qRT-PCR data by 2^−ΔΔCt^ to output histograms (figures 4 and 5; electronic supplementary material, figures S9–S11, S12c), and the two-tailed Student's *t*-tests were used to assess statistical significance (data represent mean ± s.d.; *p ≤ 0.05, **p < 0.01, ***p < 0.001).

### (e) Suppression subtractive hybridization construction, sequencing and analysis

Suppression subtractive hybridization (SSH)-D and SSH-G were constructed as previously described [32] using a CloneMiner II cDNA Library Construction Kit (Thermo Fisher Scientific, A11180) and pMD18-T Vector (TaKaRa, 6011). SSH-D has 26 100 CFUs and a recombination rate of over 96%. SSH-G has 9360 CFUs and a recombination rate of over 96%. The methods of sample dissection, RNA isolation, sequencing and bioinformatics analysis (electronic supplementary material, tables S7–S10) are same as those used for Full.

### (f) *In* *situ* hybridization

The samples were fixed in MEMPLA at room temperature for 1 h or at 4°C for 12 h, prior to storage in 75% ethanol at −20°C. The *in situ* hybridizations were performed as described in [33] and in [34] with some modifications. The templates were amplified from Full by 5′ or 3′ UTR and were inserted into pGEM-T Easy Vector Systems II (Promega, A1380), and then were amplified with M13 primers (forward: CGCCAGGGTTTTCCCAGTCACGAC; reverse: AGCGGATAACAATTTCACACAGGA). The digoxigenin (DIG)-labelled sense and antisense riboprobes were synthesized with T7 or Sp6 RNA polymerase (Roche, 11175025910) according to the manufacturer's instructions. The labelled riboprobes were precipitated with 4M LiCl/100% ethanol (40 µl/1000 µl) for 12 h. The hybridization experiment was performed in Netwell inserts (Corning, 3479). To reduce the background noise, the probe concentration was decreased to 0.1–0.2 ng µl^−1^ and the hybridization was performed at 60–65°C for 12–16 h. The post-hybridization washes were solution A (50% deionized formamide, 5× SSC and 0.1% Tween20) for 30 min × 3 at hybridization temperature, solution B (50% deionized formamide, 2× SSC and 0.1% Tween20) for 30 min × 2 at hybridization temperature, solution C (50% deionized formamide, 1× SSC and 0.1% Tween20) for 30 min × 2 at hybridization temperature, solution D (0.2× SSC and 0.1% Tween 20) for 30 min at 37°C and NaPBSTw (0.02 mol l^−1^ PBS and 0.1% Tween 20) for 10 min × 3 at room temperature on a rocker gently, with the omission of RNase treatments. The anti-DIG-AP (Roche, 11093274910) was visualized with 0.5× NBT/BCIP working solution (Roche, 11681451001) at 2–4°C for 30 min to 14 days. In order to inhibit endogenous alkaline phosphatase in the digestive tract, levamisole (Sigma, T1512) was added to NBT/BCIP working solution at a concentration of 0.48 mg ml^−1^ [35].

The samples were photographed with a stereoscopic microscope (ZEISS SteREO Discovery v. 20, Berlin, Germany). Images were combined using Adobe Photoshop CS6.

### (g) Histology

Haematoxylin–eosin stain was used as previously described [16]. The samples were photographed with a DP72 CCD on IX81 microscope (Olympus, Tokyo, Japan). Images were acquired and processed using cellSens software (Olympus, Tokyo, Japan). Images were combined using Adobe Photoshop CS6.

## 3. Results

### (a) *Branchiostoma* diverticulum epithelial cells can phagocytize food particles directly

**Figure 2.**
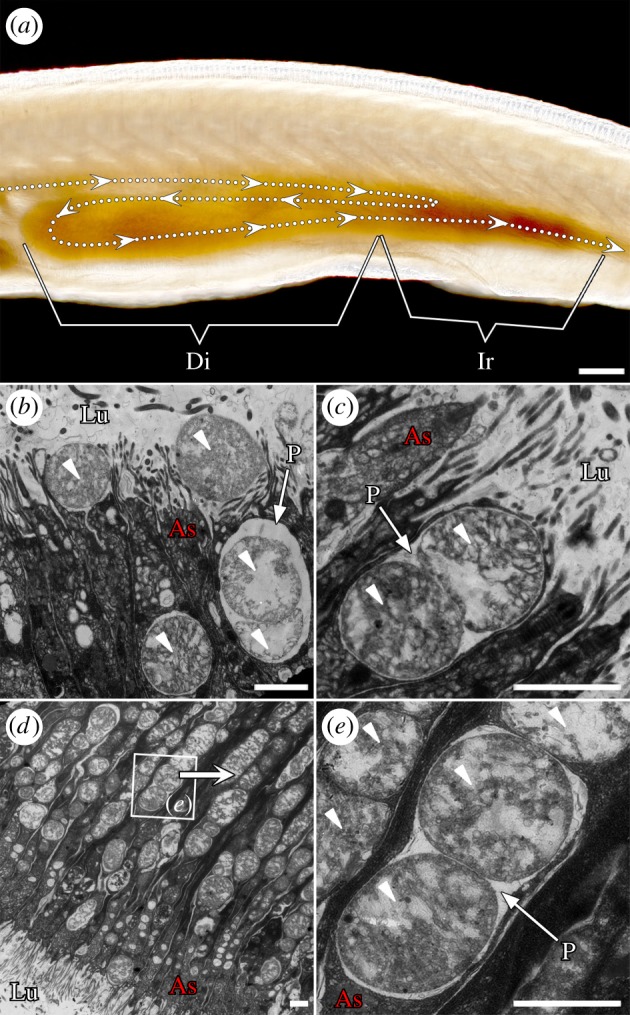
*Branchiostoma* diverticulum epithelial cells can phagocytize algal cells directly. (*a*) Food particles are transported into the diverticulum, as the arrows indicate. The ileo-colon ring sorts small and digestible cells into the diverticulum lumen. (*b*,*c*) Diverticulum epithelial cells phagocytize food cells into phagosomes directly. (*d*,*e*) Phagocytized algal cells are transported to the upper cytoplasm by phagosomes. Almost all diverticulum epithelial cells exhibit a phagocytic function (*d*). The small arrowheads mark the algal cells. Di, diverticulum; Ir, ileo-colon ring; Lu, lumen; As, apical side; P, phagosome. Scale bars: 100 µm (*a*); 2 µm (*b*–*e*).

**Figure 3.**
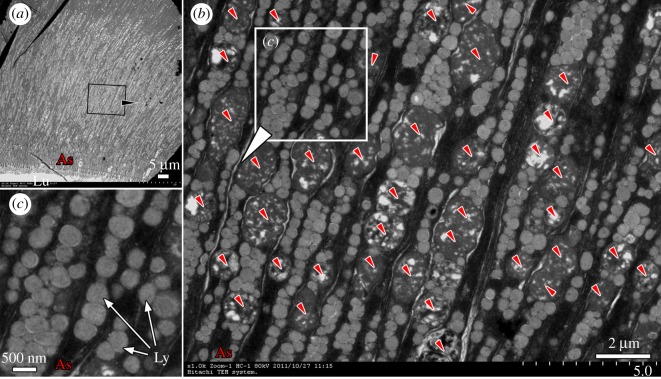
The phagocytic epithelial cells of the diverticulum produce large numbers of lysosomes to support their intracellular digestive function. (*a*) Cross section of diverticulum. (*b*, *c*) After 24 h of starvation, large numbers of lysosomes from 200 to 500 nm in diameter appear in the cytoplasm. The small arrowheads in (*b*) mark the degrading algal cells. Ly, lysosomes; Lu, lumen; As, apical side.

*Branchiostoma* filters food particles from the water with its oral cirri and pharyngeal bars. These selected food particles then flow through the oesophagus before being sorted again by the ileo-colon ring, which directs digestible food particles, such as small algal cells, to the lumen of diverticulum [36] (figure 2*a*). Interestingly, aside from being subject to normal extracellular digestion [8,10], the algal cells in the diverticulum lumen can also be phagocytized directly by diverticulum epithelial cells (figure 2*b*,*c*; electronic supplementary material, figure S1). These cells can phagocytize algal cells into their phagosomes (figure 2*c*,*e*; electronic supplementary material, figure S1c). They can even phagocytize more than one algal cell at once (figure 2*c*). The phagocytized algal cells are transported to the inner cytoplasm away from the apical side by phagosomes, waiting for further degradation (figure 2*d*,*e*; electronic supplementary material, figure S2). Importantly, the phagocytic function occurs in almost all of these cells (figure 2*d*; electronic supplementary material, figures S1c and S2). These cells produce large numbers of lysosomes to support their intracellular digestive function, especially after 24 h of starvation (figure 3).

We also discovered that, just after the larval mouth opens at about 36 h, the epithelial cells of the diverticulum primordium can immediately begin to phagocytize algal cells (electronic supplementary material, figure S3), suggesting that phagocytic intracellular digestion occurs throughout all *Branchiostoma* life stages. Additionally, the ileo-colon ring is crucial because it can control the size of the food particles that enter the diverticulum: various sizes of algal cells can be found in the region of the ileo-colon ring (electronic supplementary material, figure S4), whereas the algal cells in the diverticulum lumen are mainly around 2 µm in diameter or less (figure 2*b*-*e*; electronic supplementary material, figures S1 and S2).

### (b) The gene expression profile of diverticulum epithelial cells

In order to explore how the diverticulum epithelial cells deal with the phagocytized food particles, we constructed a full-length cDNA transcriptome library (abbreviation: Full; GenBank Accession Number: LIBEST_028542) to investigate the gene expression profile of these cells in a natural sated state (see Material and methods; electronic supplementary material, figures S5–S7). The genome of *B. belcheri* (Zhanjiang) has not been completely sequenced, and the reported genomic data from *B. belcheri* from Zhanjiang (studied in this paper), *B. belcheri* from Xiamen and *B. floridae* have obvious polymorphisms [37–40], so it is difficult to get precise transcriptome results from single NGS (next generation sequencing) techniques using non-mapping bioinformatic analysis based on the resulting shorter shotgun sequencing reads [39]. Thus, we discuss the analysis of individual intracellular digestive genes determined using the traditional Sanger method on a plasmid-based full-length cDNA library below.

The gene expression profile of the diverticulum epithelial cells overlaps with the patterns seen in both the digestive cells and phagocytes of vertebrates. The overwhelming majority of expressed gene markers belong to three families: *cathepsin*, *ferritin* and *trypsin* (table 1), and these three gene families occupy 10.57% of total ESTs and 67.79% of total annotated tissue-specific genes. The cathepsins are typical lysosomal proteases and, except for type K, most of them play major roles in intracellular protein hydrolysis [20]. In diverticulum epithelial cells, the major expressed cathepsins are *cathepsin L* and *B* (table 1), which are cysteine proteases, and *cathepsin D,* which is expressed at low levels, is aspartyl protease. Ferritin is an indispensable intracellular protein that acts as a buffer to maintain the balance of iron [41]. The intracellular iron store can prevent the spread of infective agents by impeding their proliferation [42]. The genes expressed at a moderate level are *lysozyme*, *lipase*, *VCBPs* and *carboxypeptidase Z*/*N* (table 1). Other genes expressed at low levels are *tetraspanin, legumain*, *saposin B*, *subtilisin-like protease*, *arylsulfatase B*, *Gram-negative bacteria-binding protein*, *endo-beta-1,4-glucanase*, *alpha2-macroglobulin*, *methionine adenosyltransferase*, *plasminogen*, *chitotriosidase 1-like protein*, *big defensin*, *peroxiredoxin V*, *proprotein convertase subtilisin/kexin type 1* and *Toll-interacting protein* (table 1). Among these genes, *lysozyme*, *VCBPs* [16–19], *tetraspanin, Gram-negative bacteria-binding protein*, *alpha2-macroglobulin* [24], *chitotriosidase 1-like protein* [43], *big defensin* [16] and *Toll-interacting protein* are typical immune genes, and the other genes, except for *methionine adenosyltransferase*, are the main digestive or hydrolytic genes of both digestive cells and phagocytes. The *in situ* hybridization experiments show that all the genes mentioned are expressed in diverticulum epithelial cells (electronic supplementary material, figure S8).

**Table 1.** Gene expression profile of diverticulum phagocytic epithelial cells.

gene	EST counts (per cent of total ESTs)
*cathepsin*^ab^	582 (3.89%)
*cathepsin L*	440 (2.94%)
*cathepsin B*	132 (0.88%)
*cathepsin D*	10 (0.07%)
*ferritin*^b^	504 (3.37%)
*trypsin-like serine protease*^a^	497 (3.32%)
*lysozyme*^a^^c^	112 (0.75%)
*lysozyme C*	92 (0.61%)
*lysozyme G*	20 (0.13%)
*pancreatic lipase-like protein*^a^	80 (0.53%)
*VCBPs*^c^	79 (0.53%)
*VCBP4*	58 (0.40%)
*VCBP1*	11 (0.07%)
*VCBP3*	6 (0.04%)
*VCBP5*	4 (0.03%)
*carboxypeptidase Z/N*^a^	74 (0.49%)
*tetraspanin*^c^	52 (0.35%)
*legumain*^a^^b^	47 (0.31%)
*saposin B*^a^	43 (0.29%)
*subtilisin-like protease*^a^	42 (0.28%)
*arylsulfatase B*^a^	36 (0.24%)
*gram-negative bacteria-binding protein*^c^	28 (0.19%)
*endo-beta-1,4-glucanase*^a^	25 (0.17%)
*alpha2-macroglobulin*^c^	18 (0.12%)
*methionine adenosyltransferase*	18 (0.12%)
*plasminogen*^a^	16 (0.11%)
*chitotriosidase 1-like protein*^a^^c^	11 (0.07%)
*big defensin*^c^	7 (0.05%)
*peroxiredoxin V*^b^	5 (0.03%)
*proprotein convertase subtilisin/kexin type 1*^a^	5 (0.03%)
*Toll-interacting protein*^c^	1 (0.01%)

^a^Digestive or hydrolytic genes.

^b^Genes concerned with immune reactions.

^c^Typical immune genes.

### (c) Two different gene expression states of diverticulum epithelial cells

Next, to reveal the spatiotemporal pattern of gene expression of diverticulum epithelial cells, we compare the differences in gene expression between cells in empty (after 3 days of starvation) and sated states using qRT-PCR assays. In order to determine the results of the qRT-PCR assay more precisely, we selected both 18S and cytoplasmic actin as the reference genes [44].

**Figure 4.**
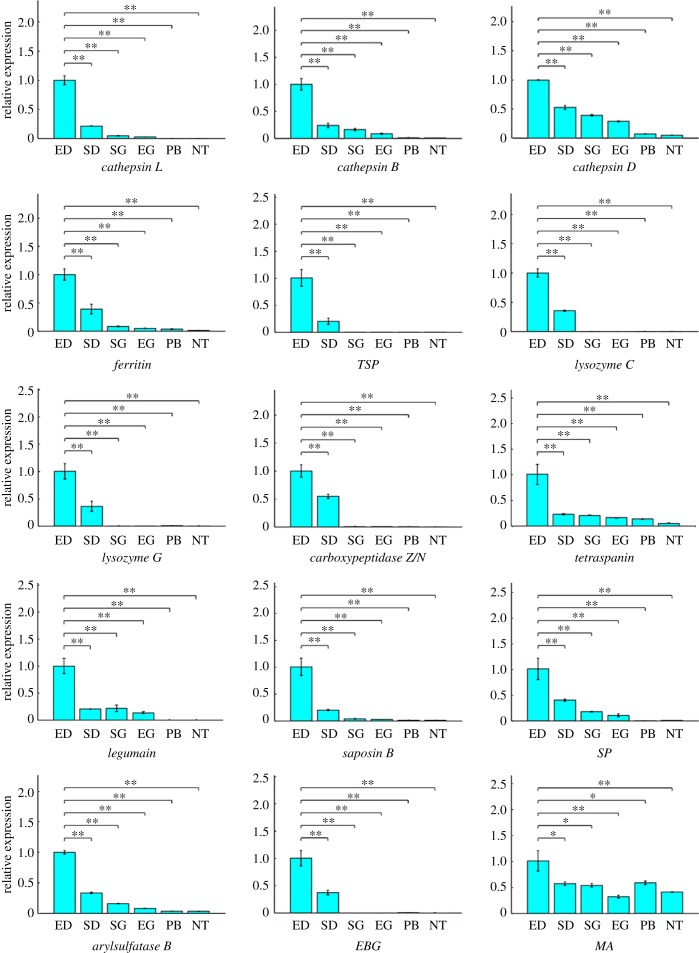
The most highly expressed genes of diverticulum phagocytic epithelial cells after 3 days of starvation. qRT-PCR results reveal that these cells accumulate endogenous digestive enzymes when starved, ensuring that they are prepared to degrade phagocytized food particles. 18S was used as the internal reference gene. Data represent mean ± s.d. Two-tailed Student's *t*-tests were used to assess statistical significance. *p ≤ 0.05, **p < 0.01, ***p < 0.001. ED, empty diverticulum; SD, sated diverticulum; SG, sated gut; EG, empty gut; PB, pharyngeal bars; NT, notochord; *TSP*, *trypsin-like serine protease*; *SP*, *subtilisin-like protease*; *EBG*, *endo-beta-1,4-glucanase*; *MA*, *methionine adenosyltransferase*.

**Figure 5.**
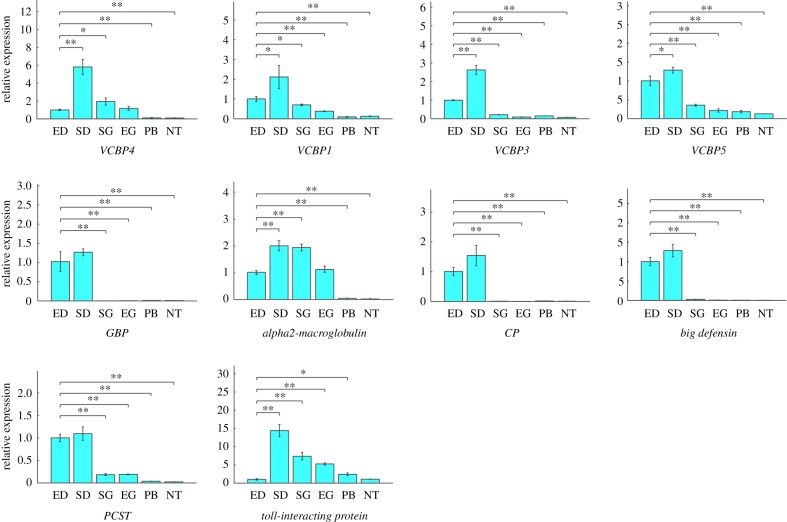
The most highly expressed genes of the diverticulum phagocytic epithelial cells in the natural sated state. The *Gram-negative bacteria-binding protein*, *alpha2-macroglobulin*, *chitotriosidase 1-like protein*, *big defensin* and *proprotein convertase subtilisin/kexin type 1* genes are highly expressed in the sated state relative to reference gene (18S). Data represent mean ± s.d. Two-tailed Student's *t*-tests were used to assess statistical significance. *p ≤ 0.05, **p < 0.01, ***p < 0.001. ED, SD, SG, EG, PB and NT: see figure 4. *GBP*, *Gram-negative bacteria-binding protein*; *CP*, *chitotriosidase 1-like protein*; *PCSP*, *proprotein convertase subtilisin/kexin type 1*.

The qRT-PCR assays show that *cathepsin L*, *B* and *D*, *ferritin*, *trypsin-like serine protease*, *lysozymes C* and *G*, *carboxypeptidase Z*/*N*, *tetraspanin*, *legumain, saposin B*, *subtilisin-like protease*, *arylsulfatase B*, *endo-beta-1,4-glucanase* and *methionine adenosyltransferase* are highly expressed in the empty state relative to both reference genes (figure 4; electronic supplementary material, figure S9). This gene expression profile indicates that the cells are prepared to degrade phagocytized food particles. It also reveals that *VCBP4*, *1*, *3*, *5*, *Gram-negative bacteria-binding protein*, *alpha2-macroglobulin*, *chitotriosidase 1-like protein*, *big defensin*, *proprotein convertase subtilisin/kexin type 1* and *Toll-interacting protein* are highly expressed in the natural sated state, relative to both reference genes (figure 5; electronic supplementary material, figure S10), indicating that phagocytized food particles can stimulate the innate immune-like response [16–19]. Moreover, our findings show that, though *pancreatic lipase-like protein*, *plasminogen* and *peroxiredoxin V* are highly expressed in diverticulum phagocytic epithelial cells, it is difficult to estimate whether they are highly expressed in either the empty or sated states because of the inconsistency caused by the two different reference genes, as one gives a high expression ratio and the other gives a low one (electronic supplementary material, figure S11). Plasminogen is the precursor of plasmin, which is a serine protease and can dissolve fibrin [45], whereas peroxiredoxin V has antioxidative and cytoprotective functions during oxidative stress triggered by immunogens [46]. The results of the qRT-PCR assays reveal that *Branchiostoma* phagocytic epithelial cells present different gene expression states in unfed versus sated states.

### (d) Gut epithelial cells can also phagocytize food particles

**Figure 6.**
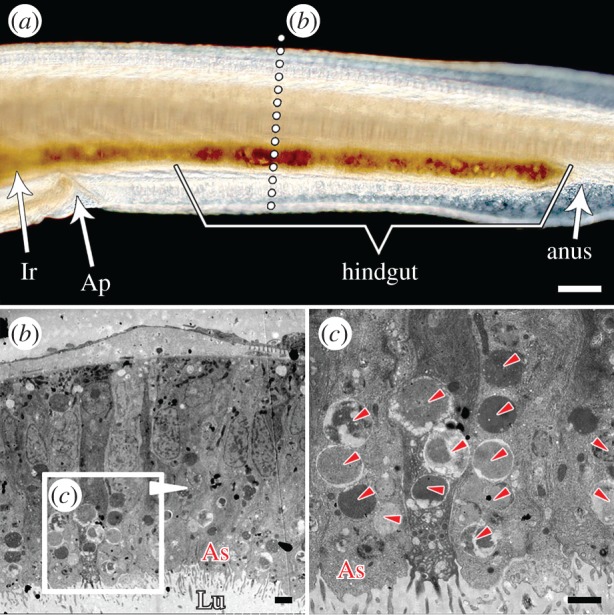
Gut epithelial cells outside the diverticulum can phagocytize. The epithelial cells in the hindgut can also phagocytize algal cells (*c*), however, the phagocytic capability of these cells seems more limited than that of diverticulum epithelial cells, which are longer and more numerous (figure 2). The small arrowheads in (*c*) mark the algal cells. Ap, atriopore; Ir, ileo-colon ring; Lu, lumen; As, apical side. Scale bars: 100 µm (*a*); 2 µm (*b*,*c*).

In addition to the epithelial cells in the diverticulum, we also found that the epithelial cells lining the hindgut can phagocytize small algal cells (figure 6). The phagocytic capability of these cells is apparently more limited than that of diverticulum epithelial cells (figure 6*b*,*c*) because of their shorter length; however, this limited intracellular digestion still improves the utilization rate of food particles. Two lines of evidence show that the diverticulum is a more specialized digestive organ than the hindgut (figure 4; electronic supplementary material, table S1). First, the qRT-PCR results in figure 4 show higher expression for many digestive and hydrolytic enzymes. In fact, among all the annotated functional genes, only *elastase I* is obviously expressed more in the gut than in the diverticulum [47] (electronic supplementary material, figure S12). Second, the suppression subtractive hybridization (SSH) results reveal that the gene expression profiles of the diverticulum and gut epithelial cells are similar, but that the diverticulum can express greater numbers of functional genes than the gut. SSH technology allows for the PCR-based amplification of the cDNA fragments that differ between control (driver) and experimental (tester) transcriptomes [32]. That is, the diverticulum-to-gut SSH (SSH-D; the tester is the diverticulum transcriptome and the driver is the gut transcriptome) contains 5182 efficient ESTs (GenBank accession number: LIBEST_028557), and the gut-to-diverticulum SSH (SSH-G; the tester is the gut transcriptome and the driver is the diverticulum transcriptome) contains 3367 efficient ESTs (GenBank accession number: LIBEST_028556).

## 4. Discussion

### (a) Food particles are exogenous immunogens to *Branchiostoma* phagocytic epithelial cells

The phagocytic epithelial cells of the *Branchiostoma* digestive tract are exposed not only to food resources, such as algal cells, but also to large quantities of exogenous, even pathogenic, single-celled organisms, such that ‘eating’ food can provoke a long-term immune-like response to protect intracellular and whole-body homeostasis. The phagocytic epithelial cells deal with this immune-like stimulation by expressing immune genes. Among these immune genes, lysozyme can present in the cytoplasmic granules to destroy Gram-positive bacteria [41], VCBPs can present an adaptive-like immune character by exhibiting high levels of polymorphism [16–19], Gram-negative bacteria-binding protein can bind to lipopolysaccharides to elicit immune responses and alpha2-macroglobulin can prevent cell lysis by inactivating various proteinases [24]. In addition, big defensin is a small cysteine-rich peptide that can bind to the cell membranes of bacteria and form pore-like defects that can lead to the lethal efflux of cytoplasmic ions and nutrients [16]. The functions of these typical immune genes and large quantities of other endogenous degrading enzymes ensure that the phagocytic epithelial cells can not only degrade phagocytized food particles sufficiently, but also maintain their cellular integrity.

### (b) The physiological function of the *Branchiostoma* diverticulum differs from that of the vertebrate liver

Ever since Müller [15], the *Branchiostoma* diverticulum has been regarded as the homologue of the vertebrate liver. However, our findings demonstrate that the cell type, tissue structure and gene expression profile of the *Branchiostoma* diverticulum are quite different from those of the vertebrate liver. Furthermore, hepatic products homologous to those in vertebrates, such as *albumin*, *alpha-fetoprotein*, *aspartate aminotransferase*, *alanine aminotransferase* and *miR-122*, were not found in the *Branchiostoma* diverticulum through the tissue-specific expressions of hallmark genes—in fact, *albumin*, *alpha-fetoprotein* and *miR-122*, are absent from the *Branchiostoma* genome [22,37–40,48]. In summary, all of the aforementioned results suggest that the main physiological functions of the diverticulum are different from those of the liver.

### (c) Phagocytic intracellular digestion in the *Branchiostoma* diverticulum cannot directly confirm the absence of liver in last common chordate

It would be reckless to infer directly that the invertebrate chordate lacked a liver just because *Branchiostoma* performs phagocytic intracellular digestion in its diverticulum. On the one hand, the determination of the homology between the diverticulum and liver needs to be supported by more evidence from evolutionary developmental biology investigations into the developmental gene regulatory networks of at least the liver, and preferably the whole endoderm [49–57]. On the other hand, because *Branchiostoma* cannot embody every feature of the ancestral chordate, and its diverticulum is possibly a specialized structure reflecting only *Brachiostoma's* own evolutionary history or lifestyle, studies of echinoderms, hemichordates, urochordates and ammocoetes (larval lampreys) are indispensable to investigating this question [58]. Furthermore, determining the evolutionary histories of animal characteristics cannot be done well without direct palaeontological evidence [59–61], so the question of whether the last common chordate had a liver or not will remain open until new fossil evidence that provides a clear answer can be found [62].

## 5. Conclusion

The results of this study confirm the existence of phagocytic intracellular digestion in *Branchiostoma* and explain why epithelial cells in the *Branchiostoma* digestive tract express a number of typical immune genes. The epithelial cells of the *Branchiostoma* digestive tract can phagocytize food particles, such as algal cells, directly. The phagocytic capability of diverticulum epithelial cells is greater than that of hind gut epithelial cells. The major physiological function of the *Branchiostoma* diverticulum is also different from that of the vertebrate liver.

## Supplementary Material

Supplementary material

## Data accessibility

The full-length cDNA transcriptome library of *Branchiostoma* diverticulum: GenBank accession number, LIBEST_028542. The diverticulum-to-gut SSH: GenBank accession number, LIBEST_028557. The gut-to-diverticulum SSH: GenBank accession number, LIBEST_028556. Data are available at: https://datadryad.org/handle/10255/3/workflow?workflowID=128155&stepID=reviewStep&actionID=reviewAction.
