# Correction to ‘Phagocytic intracellular digestion in amphioxus (*Branchiostoma*)’

**DOI:** 10.1098/rspb.2018.1277

**Published:** 2018-06-27

**Authors:** Chunpeng He, Tingyu Han, Xin Liao, Yuxin Zhou, Xiuqiang Wang, Rui Guan, Tian Tian, Yixin Li, Changwei Bi, Na Lu, Ziyi He, Bing Hu, Qiang Zhou, Yue Hu, J.-Y. Chen, Zuhong Lu

*Proc. R. Soc. B* **285**, 20180438. (Published Online 6 June 2018) (doi:10.1098/rspb.2018.0438)

The order of the last two authors in the published version was incorrect. The correct author list is:

Chunpeng He^1^, Tingyu Han^1^, Xin Liao^2,3^, Yuxin Zhou^1^, Xiuqiang Wang^4^, Rui Guan^1^, Tian Tian^5^, Yixin Li^1^, Changwei Bi^1^, Na Lu^1^, Ziyi He^6^, Bing Hu^6^, Qiang Zhou^7^, Yue Hu^1^, **Zuhong Lu**^2,3^ and **J.-Y. Chen**^1^

***to***

Chunpeng He^1^, Tingyu Han^1^, Xin Liao^2,3^, Yuxin Zhou^1^, Xiuqiang Wang^4^, Rui Guan^1^, Tian Tian^5^, Yixin Li^1^, Changwei Bi^1^, Na Lu^1^, Ziyi He^6^, Bing Hu^6^, Qiang Zhou^7^, Yue Hu^1^, **J.-Y. Chen**^2,3^ and **Zuhong Lu**^1^

Because Zuhong Lu is the first corresponding author, the **Authors for correspondence** can be kept as:

**Authors for correspondence**:

Zuhong Lu

e-mail: zhlu@seu.edu.cn

J.-Y. Chen

e-mail: chenjunyuan@163.net

