# Supplementary material for: Phagocytic intracellular digestion in amphioxus (Branchiostoma)
Source: Proc Biol Sci. 2018 Jun 6;285(1880):20180438. doi: 10.1098/rspb.2018.0438 (PMC6015868; doi:10.1098/rspb.2018.0438)
Supplement: Supplementary material [file rspb20180438supp1.docx]

**Phagocytic intracellular digestion in amphioxus (*Branchiostoma*)**

Chunpeng He, Tingyu Han, Xin Liao, Yuxin Zhou, Xiuqiang Wang, Rui Guan, Tian Tian, Yixin Li, Changwei Bi, Na Lu, Ziyi He, Bing Hu, Qiang Zhou, Yue Hu, J.-Y. Chen, and Zuhong Lu (*Proceedings of the Royal Society B*. DOI, 10.1098/rspb)

# Supplementary material

1. **Supplementary figures**
2. **Supplementary tables**
3. **Supplementary figures**

**Figure S1**

**
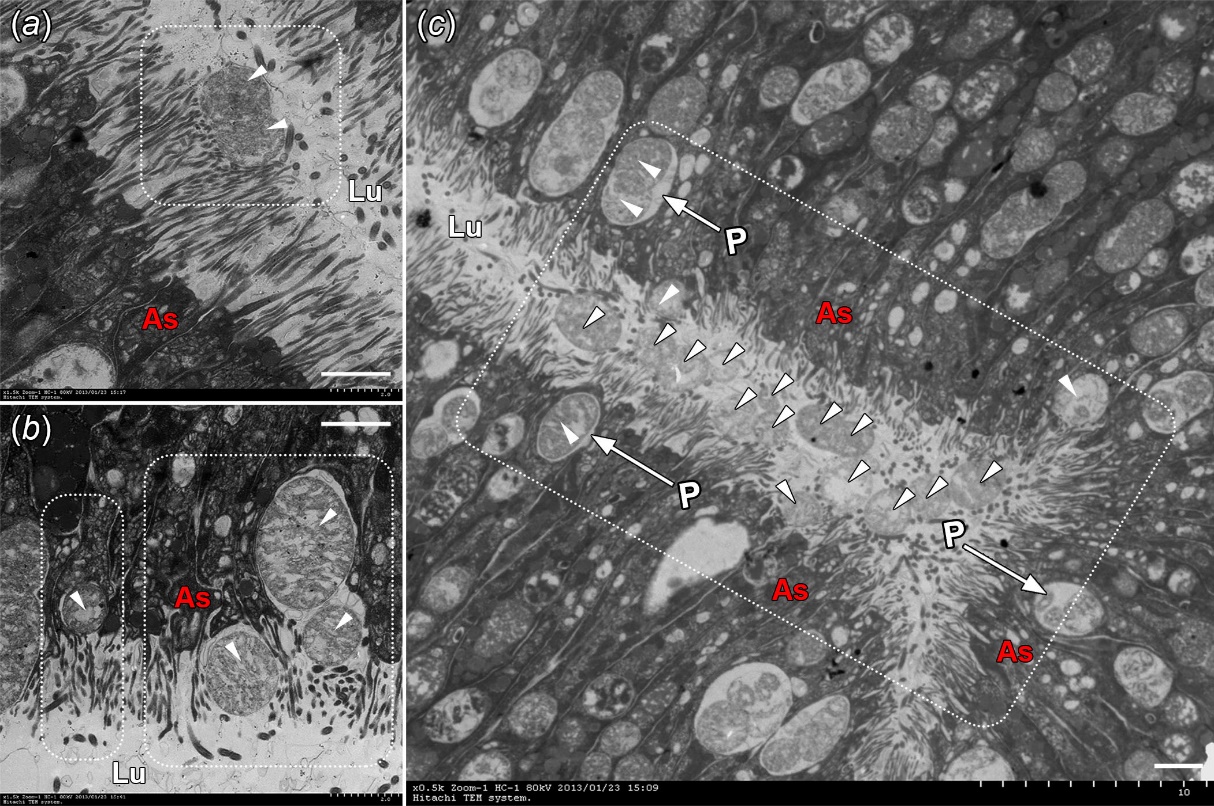
**

**Figure S1.** **Amphioxus diverticulum epithelial cells phagocytising algal cells**. (*a*–*c*) almost all diverticulum epithelial cells can phagocytise algal cells. The small arrowheads mark the algal cells. As, Lu and P, see figure 2. Scale bars: 2 μm.

**Figure S2**


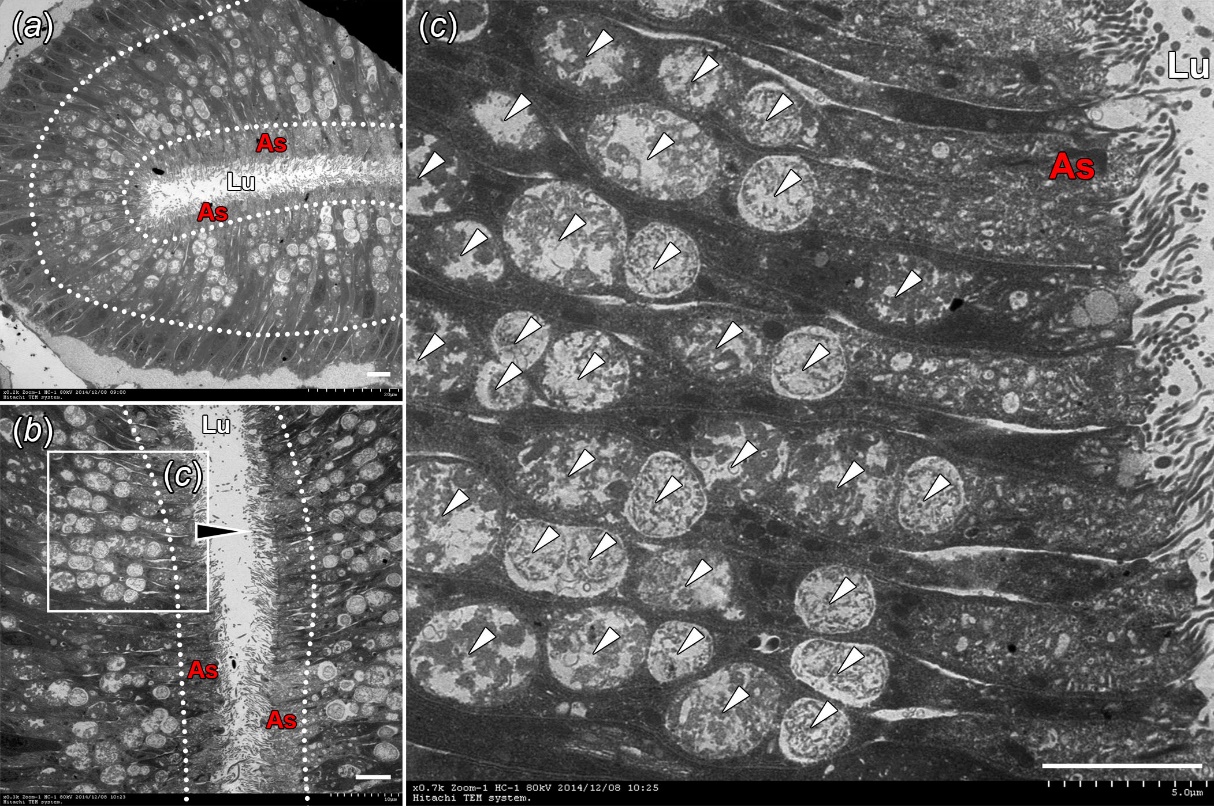


**Figure S2**. **The phagocytised food particles are transported to the upper cytoplasm to await further degradation.** (*a*–*c*) The diverticulum phagocytic epithelial cells of 2-month-old amphioxus can each phagocytise at least 5 algal cells at a time. These cells are then transported to the upper cytoplasm, where they are later degraded further (*c*). (*a*) is the caecum part of the diverticulum, and (*b*) is the body tissue. The small arrowheads in (*c*) mark the algal cells. As and Lu, see figure 2. Scale bars: 5 μm.

**Figure S3**

**
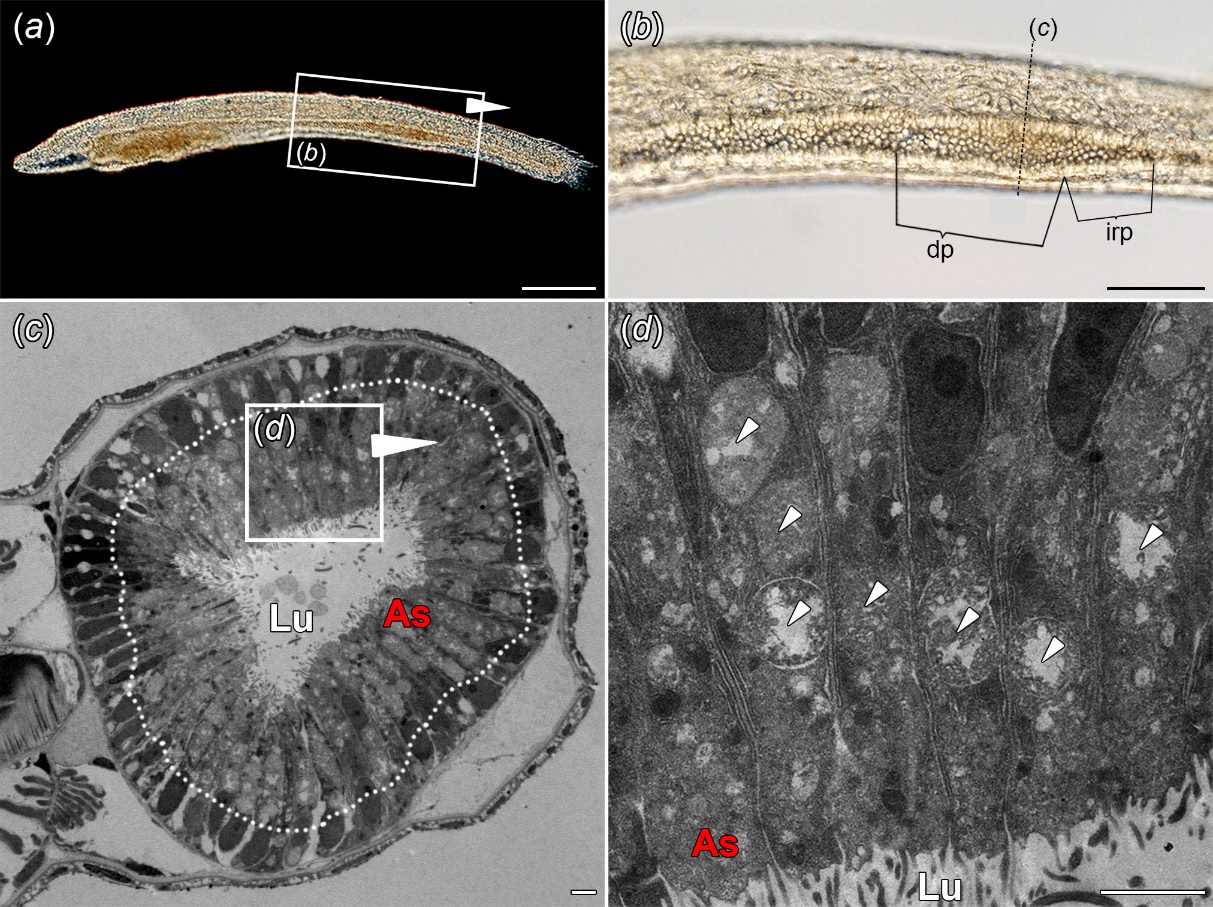
**

**Figure S3. Amphioxus uses phagocytic intracellular digestion to obtain energy throughout its life cycle.** (*a*) Amphioxus larva just after its mouth has opened (about 36 hours after fertilisation). (*b*–*d*) As soon as the larval mouth has fully opened, the phagocytic epithelial cells in the hind diverticulum primordium can phagocytise algal cells directly (*d*). The white circle in (*c*) marks algal cells in the lumen of the diverticulum primordium, and the red arrowheads in (*d*) mark the phagocytised algal cells. dp, diverticulum primordium; irp, ileo-colon ring primordium. As and Lu, see figure 2. Scale bars: 100 μm (*a*), 50 μm (*b*) and 2 μm (*c*, *d*).

**Figure S4**

**
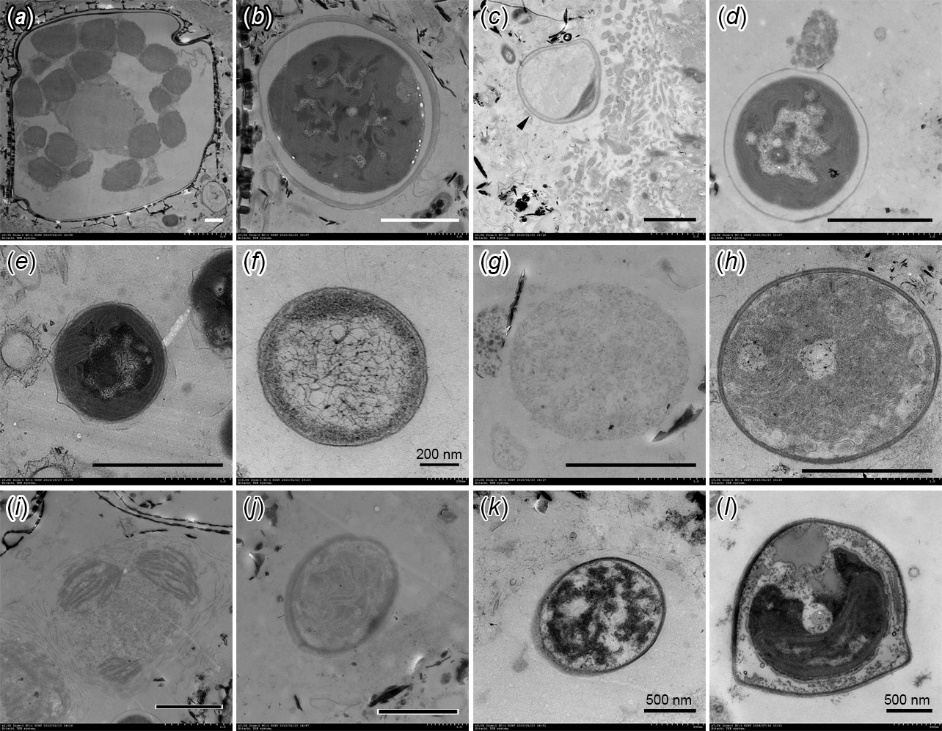
**

**Figure S4. Algal cells at the ileo-colon ring.** (*a*–*l*) Algal cells of various sizes, shapes and species can be found in the region of the ileo-colon ring; some algal cells are very large (a). Scale bars: 2 μm (*a*–*e* and *g*–*j*).

**Figure S5**


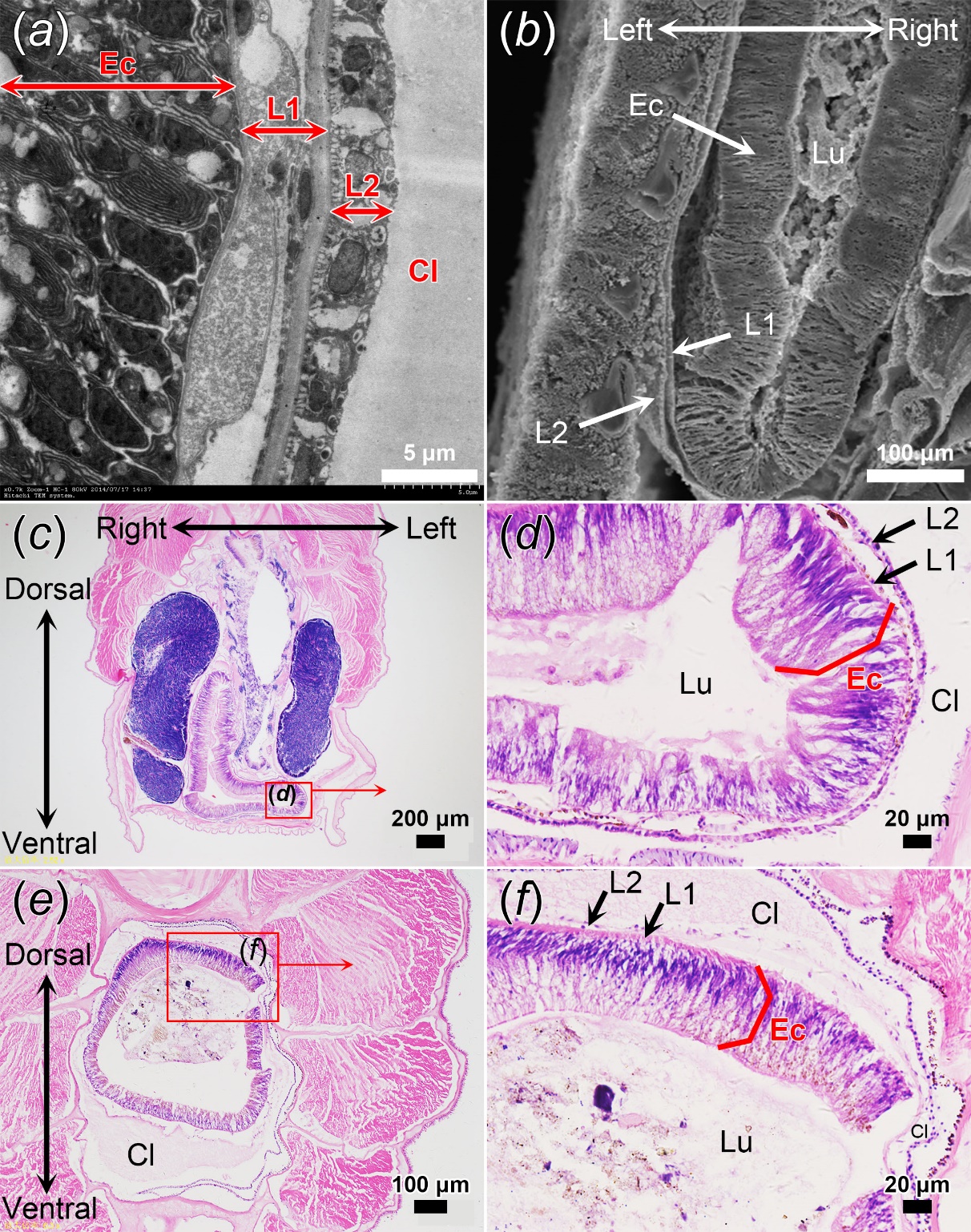


**Figure S5. The epithelium makes up the majority of diverticulum tissue mass.** (*a*–*d*) tissue components of the diverticulum. (*e*, *f*) tissue components of the hindgut. The diverticulum epithelium is a kind of pseudo-stratified columnar ciliated epithelium [9–13, 16, 21–24], and the largest tissue component of the diverticulum (*a*, *b* and *d*). In the diverticulum, two kinds of cell monolayers (L1 and 2) of connective tissue or endothelium “package” the epithelium inside and out (*a*, *b* and *d*) [9–13]. The layer cells are pavement epithelium, and one layer of L1 can connect many epithelial cells (*a*) [16, 21–24]. Thus, both the numbers and volume of epithelial cells are much larger than those of the layer cells, and the full-length cDNA transcriptome library of the diverticulum can accordingly reflect the gene expression profile of diverticulum epithelial cells. Furthermore, because the tissue components of the diverticulum and hindgut (*f*) are similar, suppression subtractive hybridization (SSH) results between the diverticulum and hindgut can also be adjusted using the gene expression results by subtracting the effect of the layer cells. Additionally, in the *Branchiostoma* digestive tract, other cell types, such as neurons, have been reported, though they are rare [36]. Ec, epithelial cell; L1, cell layer 1; L2, cell layer 2; Cl, coelom. Lu, see figure 2.

**Figure S6**

**
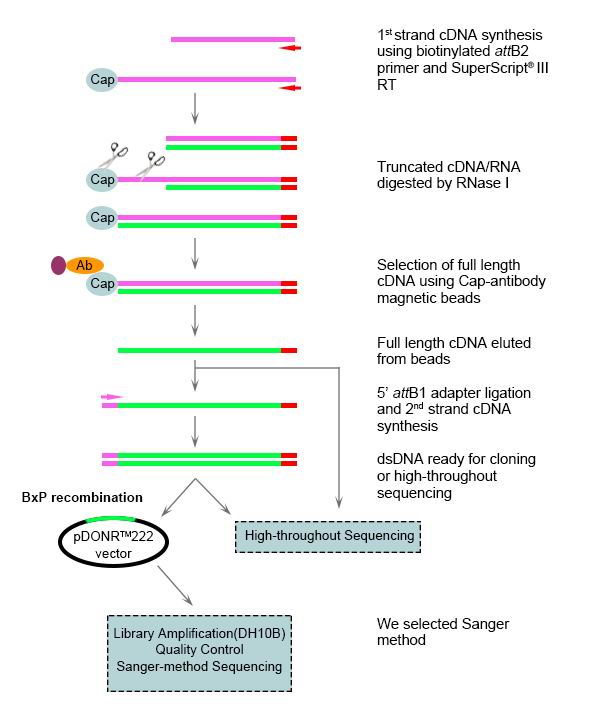
**

**Figure S6. Constructing** **the full-length cDNA library of the amphioxus diverticulum using the technical route.** Library construction can be divided into six steps. 1. Synthesise the first strand of cDNA from isolated mRNA using the SuperScript III First-Strand Synthesis System (Thermo Fisher Scientific, 18080051). In this step, single-stranded mRNA is converted into double-stranded cDNA containing *att*B sequences on each end, and PCR amplification is not required, thus avoiding the bias that may be introduced by amplification. 2. Treat the cDNA/RNA hybrids with RNase I (Thermo Fisher Scientific, EN0601). 3. Select the full-length cDNA with Cap-antibody magnetic beads and elute the-full length cDNA from the beads. 4. Ligate the 5' prime adapter to the 5’ end of cDNA, and synthesise the second strand of cDNA using the first strand of cDNA as a template. Then purify the cDNA with column chromatography to remove excess primers, adapters, and small cDNA. This step is important in the technical route, and if high-throughput sequencing technology is preferred, it will be started at this stage. 5. Perform the BP recombination reaction between the *att*B-flanked cDNA and pDONR 222. 6. Transform the BP reactions into MegaX DH10B T1 Electrocomp Cells (Thermo Fisher Scientific, C640003) by electroporation, and add freezing media to the transformed cells to get the final cDNA library. Quality control of the Full dataset involves first performing the plating assay to determine the cDNA library titre, calculating that titre from the results of the plating assay, inoculating 24 positive transformants from the plating assay randomly, and then determining the average insert size and percentage of recombinants by restriction analysis.

**Figure S7**


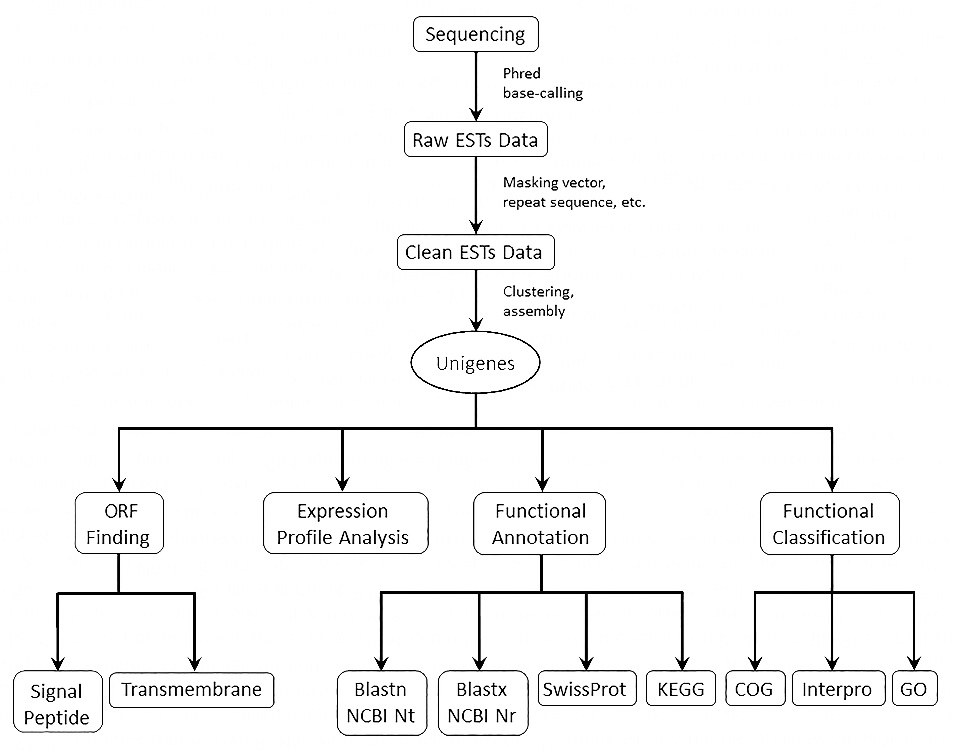


**Figure S7. Analytical method used to verify the full-length cDNA library**. The original data were processed as shown, and the unigenes were analysed by ORF finding, expression profile analysis, functional annotation, and functional classification.

**Figure S8**


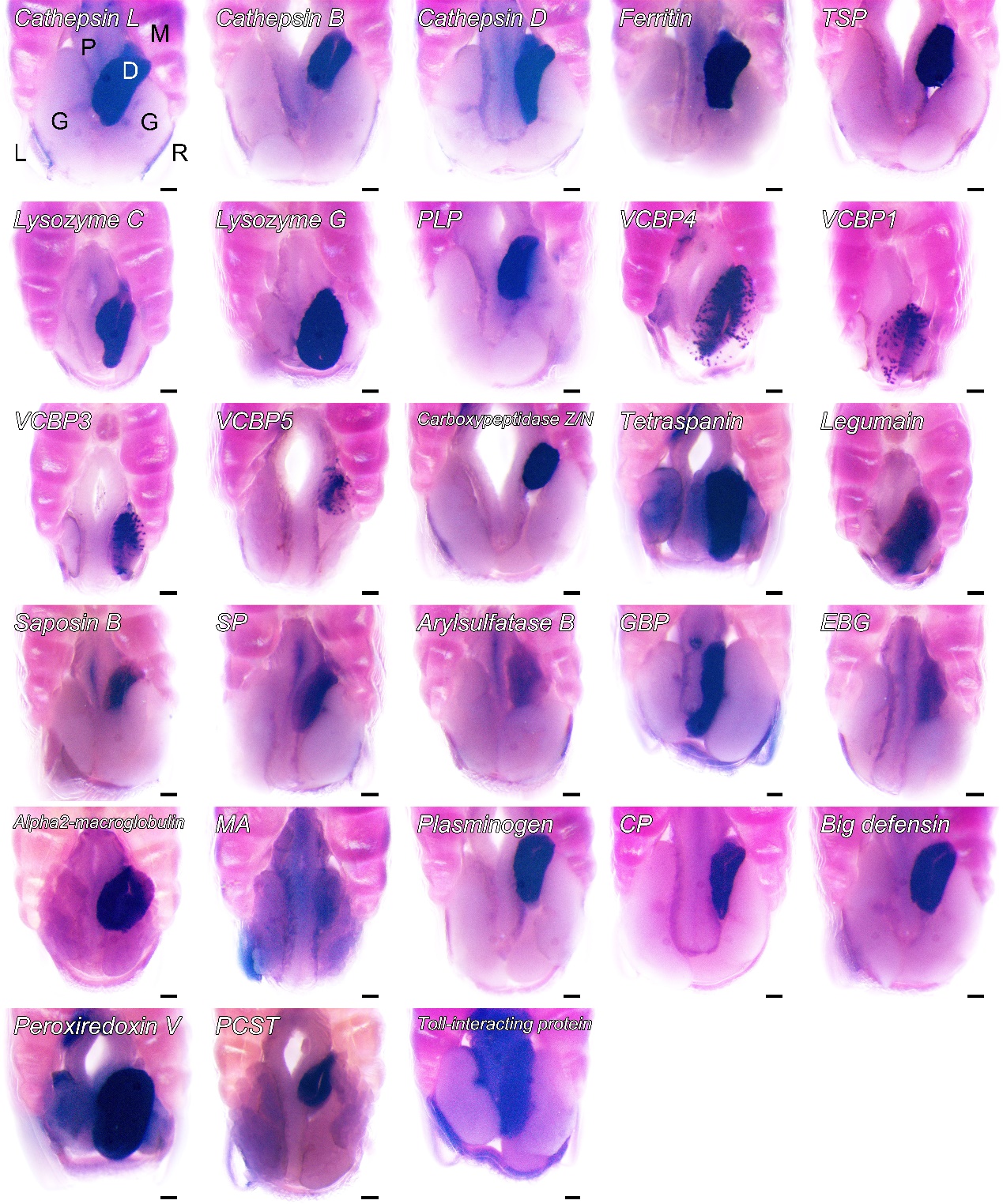


**Figure S8. All the genes in Table 1 are expressed in diverticulum epithelial cells.** *In situ* hybridization results suggest that all the genes in Table 1 are expressed in diverticulum epithelial cells. However, similar to what is seen in [16], the *VCBP*s are special and only expressed in a few cells. Whether the *VCBP*-expressing cells are a special population or whether VCBP expression can divide the epithelial cells into different groups needs more investigation. M, muscle; P, pharyngeal bars; G, gonad; D, diverticulum; L, left; R, right; TSP, SP, GBP, EBG, MA, CP and PCST, see figures 4, 5. PLP, pancreatic lipase-like protein. Scale bars: 200 μm.

**Figure S9**

**
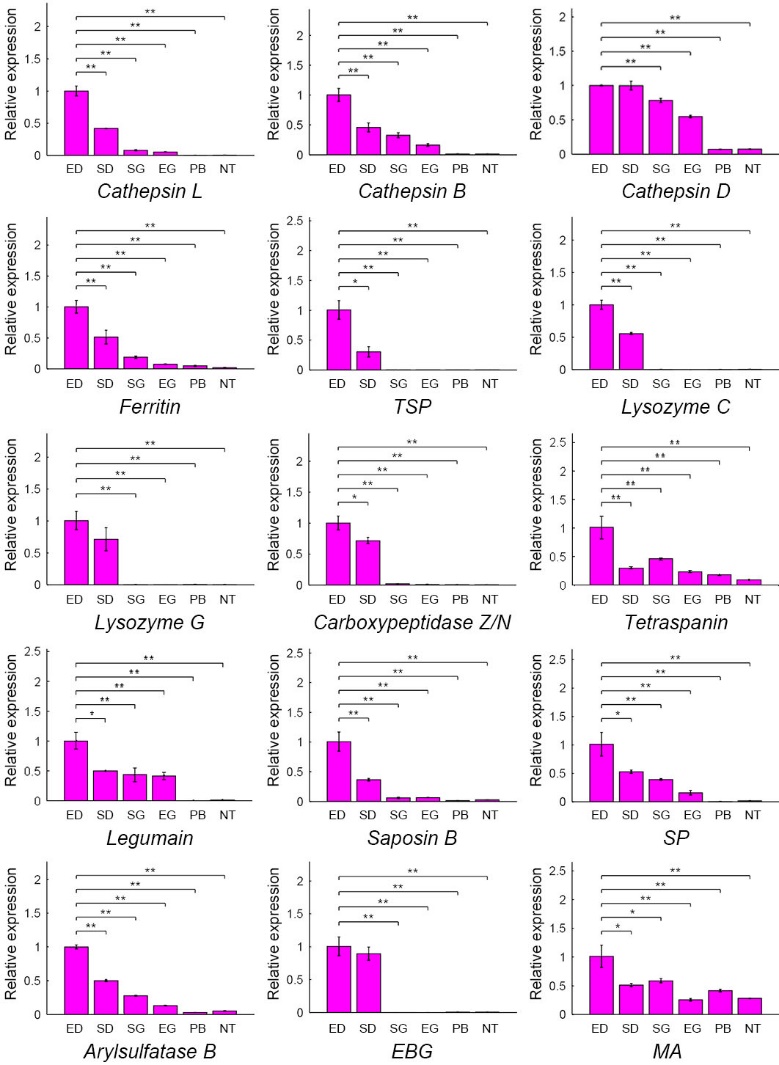
**

**Figure S9. The most highly expressed genes of diverticulum phagocytic epithelial cells after 3 days of starvation**. qRT-PCR results reveal that these cells accumulate endogenous digestive enzymes when starved, ensuring that they are prepared to degrade phagocytised food particles. Cytoplasmic actin was used as the internal reference gene. Data represent mean ± SD. Two-tailed student’s t-tests were used to assess statistical significance. * p ≤ 0.05, ** p <0.01, *** p < 0.001. ED, SD, SG, EG, PB, NT, TSP, SP, EBG, MA, see figure 4.

**Figure S10**

**
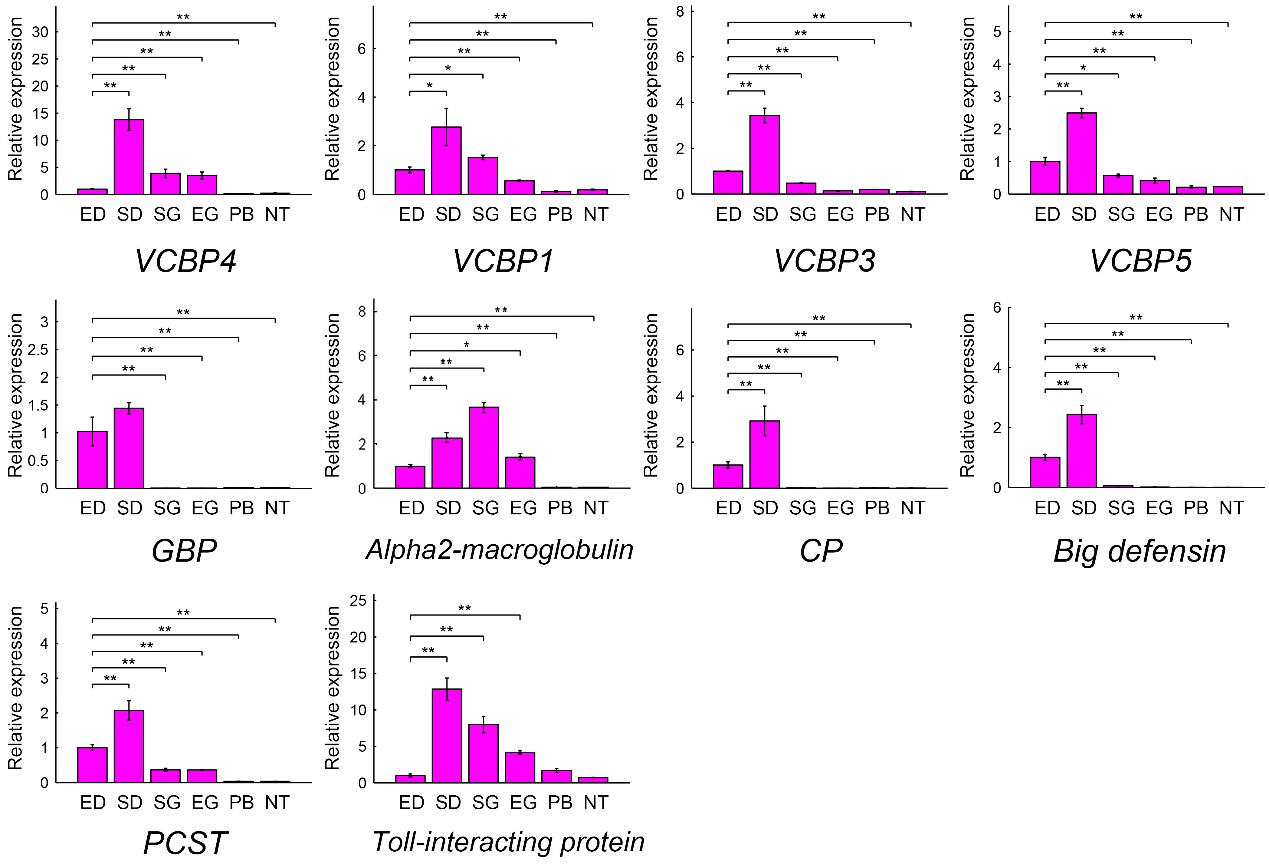
**

**Figure S10. The most highly expressed genes of the diverticulum phagocytic epithelial cells in the natural sated state**. The *Gram-negative bacteria-binding protein*, *alpha2-macroglobulin*, *chitotriosidase 1-like protein*, *big defensin* and *proprotein convertase subtilisin/kexin type 1* genes are highly expressed in the sated state relative to reference gene (cytoplasmic actin). Data represent mean ± SD. Two-tailed student’s t-tests were used to assess statistical significance. * p ≤ 0.05, ** p < 0.01, *** p < 0.001. ED, SD, SG, EG, PB and NT, see figure 4. GBP, CP and PCST, see figure 5.

**Figure S11**


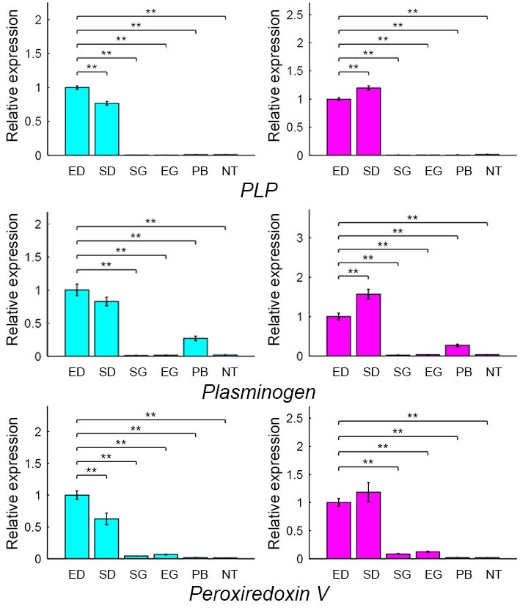


**Figure S11. Other highly expressed genes of amphioxus diverticulum phagocytic epithelial cells.** Though *pancreatic lipase-like protein*, *plasminogen* and *peroxiredoxin V* are highly expressed in diverticulum phagocytic epithelial cells, it is difficult to estimate whether they are highly expressed in empty or natural states because of the inconsistency caused by different reference genes. 18S and cytoplasmic actin were used as the internal reference genes. Cyan bars indicate gene expression relative to 18S, and magenta bars show gene expression relative to cytoplasmic actin. Data represent mean ± SD. Two-tailed student’s t-tests were used to assess statistical significance. * p ≤ 0.05, ** p < 0.01, *** p < 0.001. ED, SD, SG, EG, PB and NT, see figure 4. PLP, see figure S8.

**Figure S12**


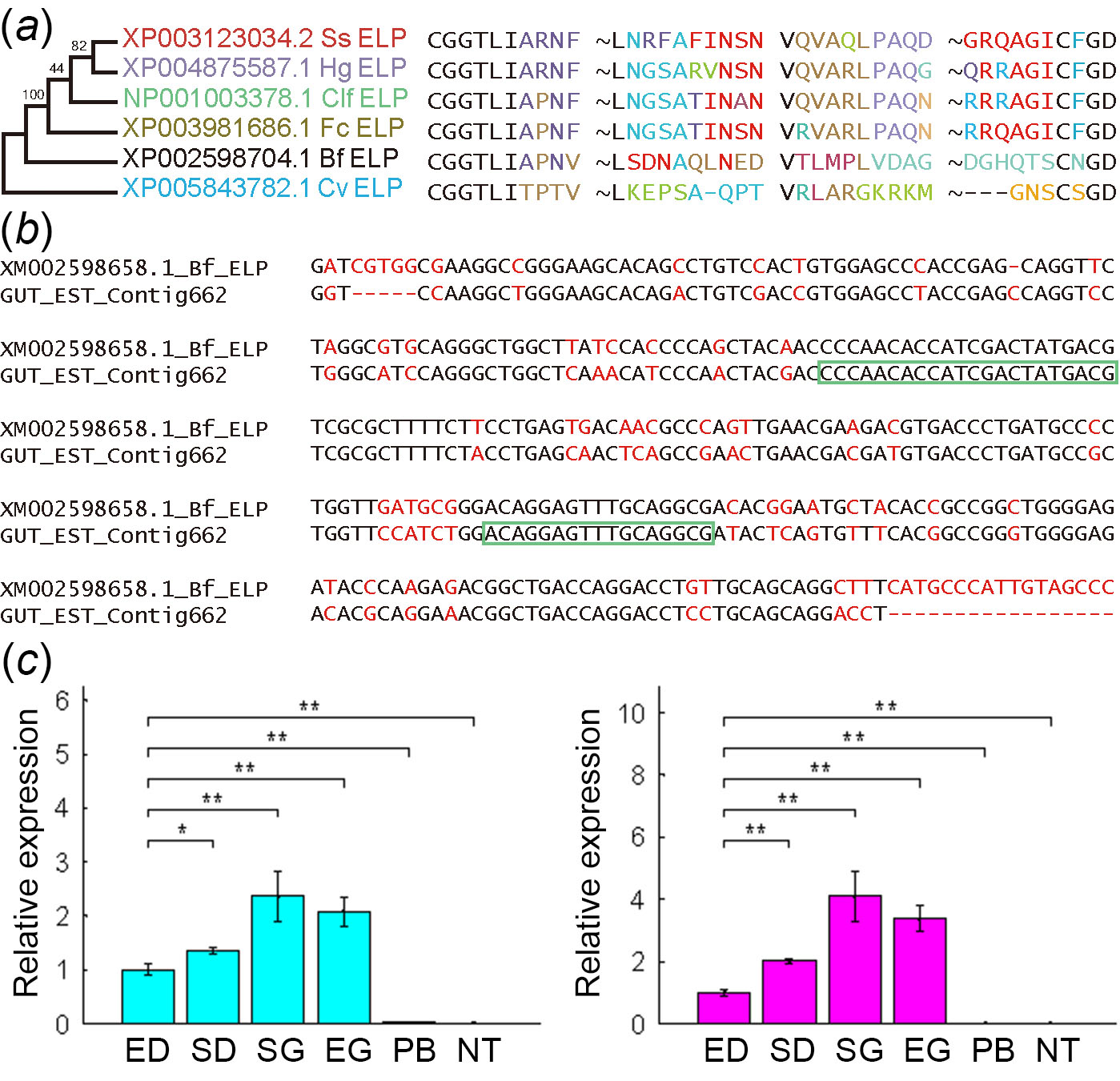


**Figure S12. The expression of *elastase I*.** (*a*) Gene identification of *elastase I*. The bootstrap consensus tree was reconstructed with MEGA6 using neighbour-joining with default settings. The values beside the branches represent the percentage of time that a node was supported over 1,000 bootstrap replications. The partially conserved domain is on the right (*a*). (*b*) Sequence alignment of the *elastase I* gene in *B. floridae* and *B. belcheri*. (*c*) qRT-PCR results show that, among all annotated functional genes, only *elastase I* is obviously expressed more in the gut than in the diverticulum. Sequences in the green boxes are the qRT-PCR primers (*b*). 18S and cytoplasmic actin were used as the internal reference genes. Cyan bars indicate gene expression relative to 18S, and magenta bars show gene expression relative to cytoplasmic actin. Data represent mean ± SD. Two-tailed student’s t-tests were used to assess statistical significance. * p ≤ 0.05, ** p < 0.01, *** p < 0.001. ED, SD, SG, EG, PB and NT, see figure 4.

1. **Supplementary tables**

**Table S1. SSH results.** SSH results show that the gene expression profiles of diverticulum and gut epithelial cells are similar, but that diverticulum cells can express more functional genes than gut cells. Among the expressed genes using SSH-G, only *elastase I* cannot be found in Full.

| **SSH-D** (NCBI Acc. No.: LIBEST 028557; total ESTs: 5,182) | | **SSH-G** (NCBI Acc. No.: LIBEST 028556; total ESTs: 3,367) | |
| --- | --- | --- | --- |
| **Genes** | EST counts  (Percent of total EST) | **Genes** | EST counts  (Percent of total EST) |
| ***Ferritin*** | 633 (12.2154%) | ***Lysozyme***  ***Lysozyme C***  ***Lysozyme G*** | 90 (2.6730%)  68 (2.0196%)  22 (0.6534%) |
| ***Lysozyme***  ***Lysozyme C***  ***Lysozyme G*** | 171 (3.2999%)  134 (2.5859%)  37 (0.7140%) | ***Elastase I-like protein*** | 74 (2.1978%) |
| ***Cathepsin***  ***Cathepsin L***  ***Cathepsin B***  ***Cathepsin D*** | 109 (2.1034%)  89 (1.7175%)  18 (0.3474%)  2 (0.0386%) | ***Ferritin*** | 72 (2.1384%) |
| ***Endo-beta-1,4-glucanase*** | 49 (0.9456%) | ***Endo-beta-1,4-glucanase*** | 52 (1.5444%) |
| ***Trypsin-like serine protease*** | 43 (0.8298%) | ***Cathepsin***  ***Cathepsin L***  ***Cathepsin B*** | 48 (1.4256%)  35 (1.0395%)  13 (0.3861%) |
| ***Saposin B*** | 26 (0.5017%) | ***Trypsin-like serine protease*** | 39 (1.1583%) |
| ***Gram-negativebacteria-binding protein*** | 15 (0.2895%) | ***Gram-negative bacteria-binding protein*** | 14 (0.4158%) |
| ***Pancreatic lipase-like protein*** | 14 (0.2702%) | ***Saposin B*** | 12 (0.3564%) |
| ***Alpha2-macroglobulin*** | 13 (0.2509%) | ***Subtilisin-like protease*** | 11 (0.3267%) |
| ***Subtilisin-like protease*** | 10 (0.1930%) | ***Alpha2-macroglobulin*** | 9 (0.2673%) |
| ***Carboxypeptidase Z/N*** | 6 (0.1158%) | ***Plasminogen*** | 3 (0.0891%) |
| ***VCBP***  ***VCBP3***  ***VCBP4*** | 6 (0.1158%)  3 (0.0579%)  3 (0.0579%) | ***Carboxypeptidase Z/N*** | 2 (0.0594%) |
| ***Chitotriosidase 1-like protein*** | 5 (0.0965%) | ***Chitotriosidase 1-like protein*** | 2 (0.0594%) |
| ***Legumain*** | 4 (0.0772%) | ***Pancreatic lipase-like protein*** | 2 (0.0594%) |
| ***Proprotein convertase subtilisin/kexin type 1*** | 4 (0.0772%) | ***Legumain*** | 1 (0.0297%) |
| ***Peroxiredoxin V*** | 3 (0.0579%) | ***Peroxiredoxin V*** | 1 (0.0297%) |
|  |  | ***Proprotein convertase subtilisin/kexin type 1*** | 1 (0.0297%) |

**Table S2. qRT-PCR primers**.

| Primer | Sequence |
| --- | --- |
| Reference gene | |
| 18S-F | TATCAGACCAAGACCAACCCG |
| 18S-R | TTTCTCAGGCTCCCTCTCCG |
| Cytoplasmic actins-F | TGGCATCATACCTTCTACAACGAG |
| Cytoplasmic actins-R | GGACAGCACGGCTTGGATG |
| Genes in Full with clustered ESTs > 15 | |
| *Methionine adenosyltransferase* | |
| Full-Contig1772-F | ACAGCGACAAGGGGTTTGACTA |
| Full-Contig1772-R | TCGTCGGTCTCGTTAGTGGC |
| *Lysozyme G* | |
| Full-Contig1793-F | ACGGAACAGGGGATAATGGG |
| Full-Contig1793-R | CCCTGTTTGATGTGCTCGGTA |
| *Legumain* | |
| Full-Contig1796-F | TTGAGGAGGCTATTCCCGTG |
| Full-Contig1796-R | CCTTTGGTGGGGTTCCTGAT |
| *Endo-beta-1,4-glucanase* | |
| Full-Contig1805-F | ATGAGGCTTTTCTTCAACGGAG |
| Full-Contig1805-R | ACTACCTGCGTCCGAAGATGC |
| *Saposin B* | |
| Full-Contig1820-F | GGGACCTGTGGGGAACTGT |
| Full-Contig1820-R | TCCAGATCAGCCTTTTCAATCA |
| *Subtilisin-like protease* | |
| Full-Contig1835-F | AAGAACCTCACGGACTACGCAG |
| Full-Contig1835-R | CGGAAGTTGTGCTCAAACAGAAT |
| *Ferritin* | |
| Full-Contig1842-F | TGACAGTTTCGTTCGTCGCA |
| Full-Contig1842-R | CTTGTTGATGCCAGCCTCG |
| *Carboxypeptidase Z*/*N* | |
| Full-Contig1847-F | ACAGCATCGCTTACAACGGA |
| Full-Contig1847-R | GAGCCAAGTGATTATCGCCC |
| *Tetraspanin* | |
| Full-Contig1848-F | GACTTCACGGGGAATGCCT |
| Full-Contig1848-R | ACTCAGGAGAACCGCAAACG |
| *Gram-negative bacteria-binding protein* | |
| Full-Contig1850-F | ACGACCCGAACAACCGAGA |
| Full-Contig1850-R | CCCAGAGCAGCCGTAGTAGC |
| *Alpha2-macroglobulin* | |
| Full-Contig1851-F | GACTACCTGTCAGCCCATCACC |
| Full-Contig1851-R | GGACAATCCAAAGAGCAACCC |
| *Trypsin* | |
| Full-Contig1867-F | AGCAGCACCACAGCCATTTC |
| Full-Contig1867-R | TGTAGTTGGGATGAACGATGACC |
| *Arylsulfatase B* | |
| Full-Contig1871-F | AAGGCGTCATCTTCAACCAGTC |
| Full-Contig1871-R | TCGGGAAGGAAGGTGAAGTTAG |
| *Pancreatic lipase-like protein* | |
| Full-Contig1883-F | TCATGCTGTTTTCGGTTCTGTC |
| Full-Contig1883-R | GAACGGGGCGATGTTAGAGA |
| *Lysozyme C* | |
| Full-Contig1886-F | GTAACTGCTGACGCTACCCTGG |
| Full-Contig1886-R | CTGCTCCAACCTCCGTGACA |
| *Cathepsin L* | |
| Full-Contig1893-F | AGAACGCCAGGCACACCAT |
| Full-Contig1893-R | GGTCTTGTTGGACGGCATAAA |
| Tissue-specific genes in Full with clustered ESTs ≤ 15% | |
| *Peroxiredoxin V* | |
| Full-Contig109-2-F | ATTCAAGCCAGAGCATACAGCA |
| Full-Contig109-2-R | ACAAAGGAATGTTCTGGGCTAAA |
| *Proprotein convertase subtilisin/kexin type 1* | |
| Full-Contig1172-2-F | GTCTGGAGAAAAGGCATCACG |
| Full-Contig1172-2-R | CCGTGCTTGTTTTCATTCGTT |
| *Big defensin* | |
| Full-Contig1258-1-F | TGCCAGATACCAAAATGGAGAAA |
| Full-Contig1258-1-R | CTCCTTCTTTGGCTTCTGTGG |
| *Chitotriosidase 1-like protein* | |
| Full-Contig1573-2-F | CGGAGAGGACAACGCTATGAAC |
| Full-Contig1573-2-R | GGACTGTTCAGACCCGTGTAGG |
| *Cathepsin D* | |
| Full-Contig1609-2-F | CGGCTTTCTGAGTGAGGATACA |
| Full-Contig1609-2-R | CACGACACCGTCCACTGAGA |
| *Cathepsin B* | |
| Full-Contig1711-2-F | CTGTTGAGGCTATGAGTGACCG |
| Full-Contig1711-2-R | ACCCGTTGCCACAGTTTTTG |
| *Plasminogen* | |
| Full-Contig1735-1-F | AGGCACCGCACGGACAT |
| Full-Contig1735-1-R | CTCAGGTCGTTGTCGGGGT |
| *VCBP4* | |
| Full-Contig1641-4-F | CGTCACATGCCTGTAAATGG |
| Full-Contig1641-4-R | AAGTTACGCCCGTGATTGTC |
| *VCBP1* | |
| Full-Contig1611-F | ATCCACGTCACAGCCTAACC |
| Full-Contig1611-R | CCTTAAACCAGGCGATGGTA |
| *VCBP3* | |
| Full-Contig1311-F | ACAGTCCGTACCACCCAGAC |
| Full-Contig1311-R | CGCCGAAACTCTCCTTGTAG |
| *VCBP5* | |
| Full-Contig832-F | CGTCTGTCCGTCGTTAACCT |
| Full-Contig832-R | GAGCCATATCCATCCGAAGA |
| Toll-interacting protein | |
| JZ816752-F | AACCCTCGCTGGAATAAGGT |
| JZ816752-R | GTACCAGTCGTCCACCGTCT |

**Tables S3–S10**

Table S3_The total information of Full, Table S4_The total statistics of Full, Table S5_Fasta_The assembling results of Full, Table S6_Fasta_GenBank Accession and Clone Id of Full, Table S7_The total_Information of SSH-D, Table S8_The total_Statistic of SSH-D, Table S9_The total_Information of SSH-G, and Table S10_The total_Statistic of SSH-G have all been deposited in: <https://datadryad.org/handle/10255/3/workflow?workflowID=128155&stepID=reviewStep&actionID=reviewAction>, from which the documents can be freely downloaded.
